# TUNING ENVIRONMENTAL LIGHTING TO IMPROVE SLEEP QUALITY AND COGNITIVE PERFORMANCE IN OLDER ADULTS

**DOI:** 10.1093/geroni/igz038.2443

**Published:** 2019-11-08

**Authors:** Nastaran Shishegar, Mohamed Boubekri

**Affiliations:** University of Illinois at Urbana-Champaign, Champaign, Illinois, United States

## Abstract

Sleep is important for memory consolidation, hence the disruption of normal sleep patterns as a result of age-related changes in the circadian system could be one of the contributors to memory impairment among older adults. It is now well-established that light is the main environmental element that synchronizes circadian rhythms. An appropriate lighting condition can be considered as a non-pharmacological solution to improve the sleep quality of individuals and consequently their overall health and well-being. The present study investigates the effectiveness of two proposed whole-day lighting interventions (L1 and L2) applied by Tunable White Lighting Technology (TWLT) on sleep quality and cognitive performance in older adults. Both lighting interventions provide a high illuminance level (500 lux) in the morning and then the illumination is dimmed gradually throughout the day and reached 100 lux in the evening. However, while L1 offers a constant Correlated Color Temperature (CCT) of 2700K, during the L2 intervention, the CCT is changing in the range of 6500K – 2700K from morning towards evening. Fifteen healthy older adults (mean age = 73.2 years; 12F) participated in a 41-day counterbalanced crossover study. Participants were exposed to each lighting condition for 9 days. Actigraphy, standard questionnaires (PROMIS and PSQI), and tests (Trail Making Test (TMT) A & B and Digit Symbol Substitution Test (DSST)) were employed to measure sleep quality and cognitive performance before, during, and after lighting interventions. Significant improvements in sleep quality and cognitive performance were found for both lighting interventions with better outcomes for L2.

